# Combined oral contraceptive pill-exposure alone does not reduce the risk of bacterial vaginosis recurrence in a pilot randomised controlled trial

**DOI:** 10.1038/s41598-019-39879-8

**Published:** 2019-03-05

**Authors:** Lenka A. Vodstrcil, Ms Erica Plummer, Christopher K. Fairley, Gilda Tachedjian, Matthew G. Law, Jane S. Hocking, Ms Karen Worthington, Ms Mieken Grant, Nita Okoko, Catriona S. Bradshaw

**Affiliations:** 1 Central Clinical School, Monash University, Melbourne, 3004 Australia; 2 Melbourne Sexual Health Centre, Alfred Hospital, Carlton, 3053 Australia; 3 Melbourne School of Population and Global Health, University of Melbourne, Parkville, 3010 Australia; 4 Burnet Institute, Melbourne, 3004 Australia; 5 Department of Microbiology, Monash University, Clayton, 3168 Australia; 6 Department of Microbiology and Immunology, University of Melbourne, at the Peter Doherty Institute of Infection and Immunity, Melbourne, 3000 Australia; 7 School of Science, College of Science, Engineering and Health, RMIT University, Melbourne, 3000 Australia; 8 Kirby Institute, University of New South Wales, Kensington, 2052 Australia

## Abstract

We conducted a pilot open-label randomised controlled trial of combined (oestrogen-progesterone) oral contraceptive pill (COCP)-exposure aimed to examine its effect on BV-recurrence following first-line antibiotics compared to antibiotics alone. Ninety-five women with symptomatic BV were prescribed antibiotic therapy, randomised to COCP-exposure (intervention) or current non-hormonal contraceptive practices (control) and followed monthly for six-months or until BV-recurrence. Modified intention-to-treat methods requiring either ≥1 clinical (primary/Amsel-outcome) or ≥1 microbiological (secondary/Nugent-outcome) BV-recurrence assessment were applied to determine cumulative recurrence rates. Secondary Cox regression analyses assessed factors associated with recurrence in all women. 92/95 women randomised provided baseline requirements. BV-recurrence rates were similar in women randomised to the COCP (primary/Amsel-outcome: 10/100PY, 95%CI: 6,19/100PY) compared to controls (14/100PY, 95%CI: 9, 21/100PY, p = 0.471). In secondary analyses sex with the same pre-treatment regular sexual partner (RSP; Amsel: Adjusted Hazard Ratio [AHR] = 3.13, 95%CI: 1.41, 6.94, p = 0.005; Nugent: AHR = 2.97, 95%CI: 1.49, 5.83, p = 0.002) and BV-history (Amsel: AHR = 3.03, 95%CI: 1.14, 6.28; Nugent: AHR = 2.78, 95%CI: 1.22, 6.33) were associated with increased BV-recurrence. This pilot RCT of COCP-exposure did not improve BV cure but found sex with an RSP and BV-history were associated with recurrence, although impacted by sample size and attrition. These data indicate reinfection from an untreated RSP and persistence of BV-associated bacteria are integral to the pathogenesis of recurrence and may overwhelm potential beneficial effects of hormonal contraception on the vaginal microbiota.

## Introduction

Bacterial vaginosis (BV) is the most common vaginal dysbiosis. Heterogeneous diverse bacteria dominate^1–3^ and protective^4–6^ *Lactobacillus* spp. are depleted, leading to a compositional shift in the vaginal microbiota. Although first-line antibiotics^7,8^ have equivalent one-month cure rates of 70–80%^9^, six-month recurrence rates >50% ensue^10,11^. Given the global burden and morbidity associated with BV^12^, there is a pressing need to improve treatment efficacy to reduce sequelae.

Hormonal contraceptive use, predominantly reflecting combined (oestrogen-progesterone) oral contraceptive pill (COCP)-exposure, is associated with significantly reduced BV prevalence (pooled effect size [pES] = 0.68, 95%CI: 0.63,0.73), incidence (pES = 0.82, 95%CI: 0.72,0.92), and recurrence (pES = 0.69, 95%CI: 0.59,0.91) by meta-analysis^13^. However this effect may be due to confounding factors influencing contraceptive choices, including partner type (ongoing/regular sexual partner or short-term partner/s). We aimed to determine by randomised controlled trial (RCT) if COCP-exposure following antibiotic therapy reduces BV-recurrence risk within six-months, compared to antibiotic therapy alone. We hypothesized sustained exogenous sex-hormone exposure may support a healthy vaginal microbiota and reduce recurrence rates. This pilot trial was initially powered for a fully-funded RCT, with ongoing funding expected. The primary objective was to obtain efficacy estimates of the impact of COCP-exposure on recurrence rates and to establish feasibility using the following parameters; recruitment, adherence, adverse effects, and retention. Funding for the full-RCT was not secured and the pilot was terminated without viewing the data, and analyses were then performed.

We conducted modified intention-to-treat (mITT) analyses of women who returned for ≥1 clinical assessment (primary/Amsel-outcome) or returned ≥1 sample for microbiological assessment (secondary/Nugent-outcome) of BV-recurrence within six-months of antibiotics. Secondary analyses assessed characteristics associated with BV-recurrence in all women.

## Results

### Participant flow, numbers analysed

**Figure 1:**
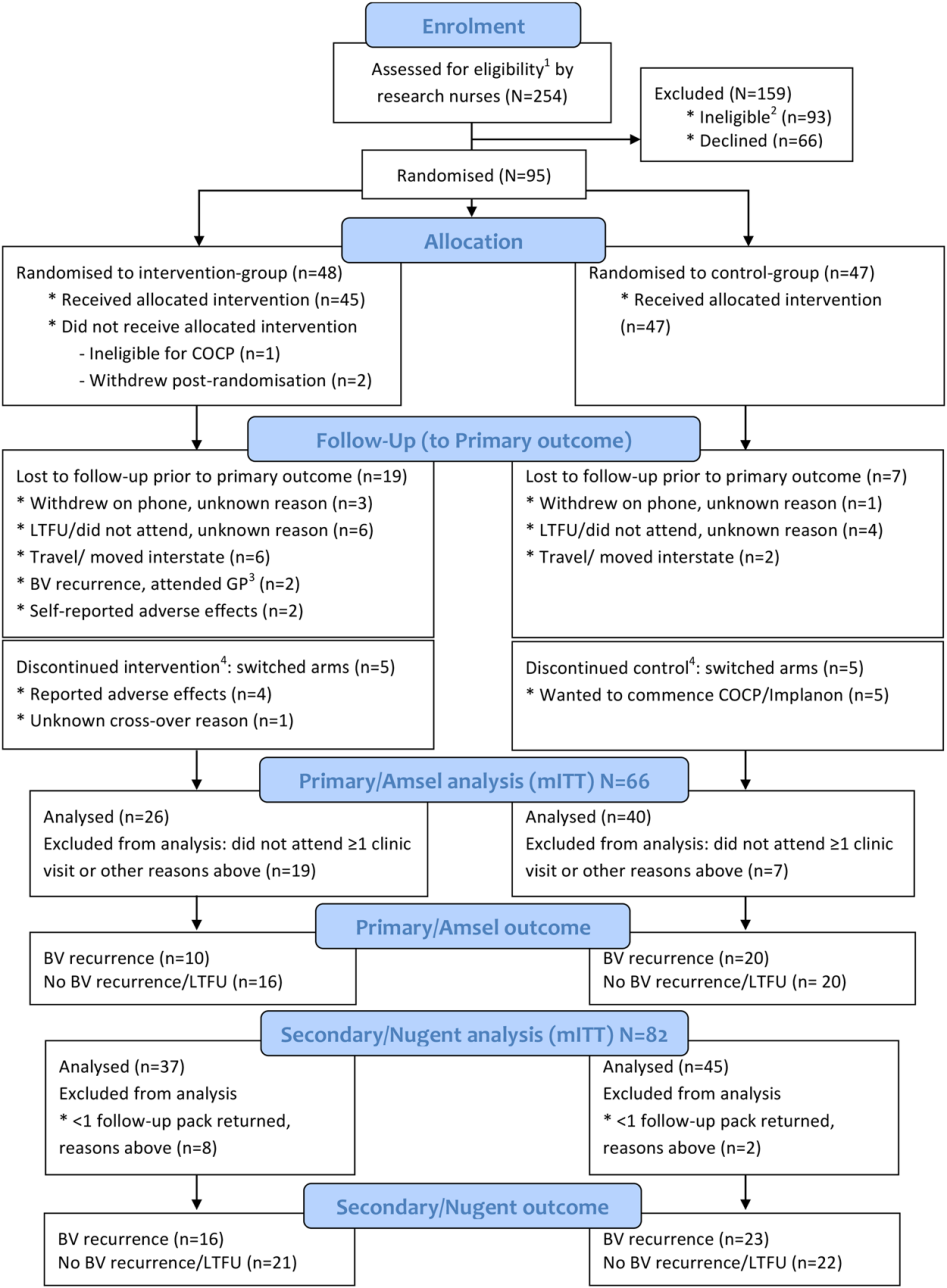
Participant flow through the study. CONSORT Diagram of the participant population. Abbreviations: COCP, combined oral contraceptive pill; LTFU, loss-to-follow-up; GP, General Practice; mITT, modified intention-to-treat. ^1^women with symptomatic BV were eligible if they were 18–45 years of age; ^2^women were ineligible if they were planning substantial travel during follow-up, were not equally comfortable being randomised to the COCP or remaining with their current contraceptive practice, were already using a hormonal contraceptive, had contraindications to the COCP^43^ or were pregnant/wanted to become pregnant; ^3^included in secondary/Nugent-outcome analyses; ^4^relevant for per protocol analysis.

From July 2014-March 2016, there were 1644 consultations in which BV was diagnosed, representing 612 women. Clinicians did not refer women to trial nurses if they were known to be ineligible, wanted/did not want to commence hormonal contraception, or declined referral. Of 254 women referred to the nurse for eligibility assessment, 95 (37%, 95%CI: 31,44) were recruited, 93 (37%) were ineligible and 66 declined participation (26%, Fig. 1).

Two of 95 who consented withdrew immediately post-randomisation and another was found to have a COCP-contraindication; all three were excluded from analyses (no baseline data). The remaining 92 provided baseline characteristics (Table 1), and completed home-packs/clinic visits until primary or secondary outcome, withdrawal or LTFU (Table 2). Baseline characteristics were comparable between groups except more in the intervention-arm reported ≥15 lifetime male partners (71% *vs* 47% controls).

**Table 1:** Baseline characteristics in women providing baseline data (N = 92).

Characteristic	Total (N = 92)	Control (N = 47)	COCP (N = 45)
	n (%^a^)	n (%^a^)	n (%^a^)
Age (Median, Range)	27 (20–46)	27 (20–42)	27 (20–46)
<28 y	55 (60)	28 (60)	27 (60)
≥28 y	37 (40)	19 (40)	18 (40)
**Country of birth**			
Australia/New Zealand	43 (47)	20 (43)	23 (51)
Other^b^	49 (53)	27 (57)	22 (49)
**Education level**			
Up to Year 12/other certificates/diplomas	18 (20)	11 (23)	7 (16)
Tertiary/Masters/PhD	74 (80)	36 (77)	38 (84)
**Past history of BV**			
No	26 (29)	13 (28)	13 (29)
Yes	65 (71)	33 (72)	32 (71)
**Hormonal contraceptive use in prior 6mo** ^**c**^			
No	73 (78)	38 (81)	35 (76)
Yes	20 (22)	9 (19)	11 (24)
**Current douching**			
No	78 (86)	40 (85)	38 (86)
Yes	13 (14)	7 (15)	6 (14)
**Douching frequency**			
None	78 (86)	40 (85)	38 (86)
Weekly or less often	7 (8)	3 (6)	4 (9)
Daily	6 (7)	4 (9)	2 (5)
**Smoking**			
Non-smoker	46 (50)	24 (51)	22 (49)
1–34 cigarettes per week	20 (22)	11 (23)	9 (20)
≥35 cigarettes per week	26 (29)	12 (26)	14 (31)
**No. of lifetime MSPs**			
<15	38 (42)	25 (53)	13 (29)
≥15	54 (58)	22 (47)	32 (71)
**No. of lifetime FSPs**			
none	60 (65)	31 (66)	29 (64)
≥1	32 (35)	16 (34)	16 (36)
**Current RSP at baseline**			
No	44 (48)	23 (49)	21 (47)
Yes	48 (52)	24 (51)	24 (53)
**Sex of RSP at baseline**			
Male	41 (85)	19 (79)	22 (92)
Female	7 (15)	5 (21)	2 (8)
**Current sex work**			
No	81 (88)	42 (89)	39 (87)
Yes	11 (12)	5 (11)	6 (13)
**Sex practices in previous 3 months**			
**No. of MSPs in previous 3 mo**			
<2	42 (46)	23 (49)	19 (42)
≥2	50 (54)	24 (51)	26 (58)
**No. of FSPs in previous 3 mo**			
none	75 (82)	35 (74)	40 (89)
≥1	17 (18)	12 (26)	5 (11)
**Any vaginal intercourse**			
No	12 (13)	10 (21)	2 (4)
Yes	80 (87)	37 (79)	43 (96)
**Condom-use for vaginal intercourse**			
Always	19 (24)	10 (27)	9 (21)
Not always	61 (76)	27 (73)	34 (79)
**Any receptive oral sex**			
No	19 (21)	10 (22)	9 (20)
Yes	72 (79)	36 (78)	36 (80)
**Any receptive anal intercourse**			
No	77 (84)	40 (85)	37 (82)
Yes	15 (16)	7 (15)	8 (18)
**Condom-use for anal intercourse**			
Always	2 (13)	2 (29)	0
Not always	13 (87)	5 (71)	8 (100)

Continuous variables were dichotomised at median value.

No., number; y, year; mo, months; RSP, regular sexual partner; MSP, male sexual partner; FSP, female sexual partner.

^a^Up to 2% of participants may have missing data for some variables; therefore, proportions are calculated using available data.

^b^Other country of birth comprised predominately of individuals from Britain and Ireland (31%), China, Taiwan and South East Asia (20%), and Eastern and Western Europe (16%).

^c^Reflects any method of hormonal contraceptive use in the 6 mo prior to enrolment. Women reported prior implant (n = 2, removed > 2 mo ago), ring (n = 2, removed > 4 mo ago), injection (n = 2, > 3 mo ago), Mirena® (n = 1, removed > 4 mo ago) or COCP-use (n = 13, stopped > 2 mo ago).

**Table 2:** Participant numbers and randomisation summary.

		Study Arm	
	Total	Control	COCP
	N	n (%)	n (%)
**Baseline data**			
Provided baseline data	92	47 (51.1)	45 (48.9)
**BV treatment prescribed**			
Metronidazole po	77 (83.7)	39 (83.0)	38 (84.4)
Clindamycin pv	15 (16.3)	8 (17.0)	7 (15.6)
**COCP prescribed**			
30 mcg EE, 150 mcg LNG			41 (91.1)
30 mcg EE, 3 mg DPN			2 (4.4)
20 mcg EE, 3 mg DPN			1 (2.2)
20 mcg EE, 100 mcg LNG			1 (2.2)
***Longitudinal data***			
**Modified ITT ≥ 1intervals of FU provided** ^**a**^			
Primary/Amsel-outcome	66 (71.7)	40 (85.1)	26 (57.8)
Secondary/Nugent-outcome	82 (89.1)	45 (95.7)	37 (82.2)
**Per protocol analysis ≥ 1 intervals of FU provided** ^**b**^			
Primary/Amsel-outcome	65 (68.4)	40 (85.1)	25 (55.6)
Secondary/Nugent-outcome	81 (88.0)	45 (95.7)	36 (80.0)
Pill exposure analysis ≥ 1 intervals of FU provided^c^	***N intervals***	***Off COCP***	***On COCP***
Primary/Amsel-outcome	299	169	130
Secondary/Nugent-outcome	334	184	150
Cross-over study arms prior to Amsel-outcome^d^	***N = 66***	***N = 40***	***N = 26***
No	56 (84.8)	35 (87.5)	21 (80.8)
Yes	10 (15.2)	5 (12.5)	5 (19.2)
Cross-over study arms prior to Nugent-outcome^d^	***N = 82***	***N = 45***	***N = 37***
No	72 (87.8)	40 (88.9)	32 (86.5)
Yes	10 (12.2)	5 (11.1)	5 (13.5)

COCP, combined oral contraceptive pill; BV, bacterial vaginosis; po, orally; pv, vaginally; EE, ethinyloestradiol; LNG, levonorgestrel; DPN, drospirenone; mg, milligrams; mcg, micrograms; Amsel, Amsel criteria used for diagnosis of BV;^41^ Nugent, Nugent scoring used for diagnosis of BV;^42^ FU, follow-up.

^a^Modified Intention-to-treat (ITT): provided at least one interval of follow-up (Amsel or Nugent), analysis censored at BV diagnosis or loss-to-follow-up (LTFU).

^b^Per protocol analysis: provided at least one interval of follow-up, analysis censored at BV diagnosis, change of treatment-arm (cross-over^d^) or LTFU.

^c^Pill exposure analysis: analysed according to whether on or off the COCP for any given interval, presented for at least one interval of follow-up, censored at BV diagnosis or LTFU.

^d^Cross-over study arms prior to outcome used to inform Per protocol analysis.

### Adherence and adverse effects

#### Antibiotic treatment: adherence and adverse effects - day 8

The majority of women eligible for mITT analyses (N = 82) were prescribed 7-days of oral metronidazole (n = 67, 82%, 95%CI: 72,89) with 15 (18%, 95%CI: 10,28) prescribed 7-days of clindamycin (Table 2). Of those prescribed metronidazole, 57 completed all 14 doses, seven took 11–13 doses and one only six doses. Thirteen of 14 women prescribed clindamycin completed all seven doses, with one using four doses. Four (three prescribed metronidazole and one clindamycin) did not return adherence data.

Twenty-six of 81 women (32%, 95%CI: 22,43) reported possible antibiotic adverse effects including headache/nausea/fatigue (n = 9), mood change (n = 1), mild gastrointestinal symptoms (n = 5), a metallic taste in the mouth (n = 1), thrush/thrush-like symptoms (n = 6), itchiness (n = 4) and bleeding/spotting (n = 2).

#### COCP-exposure: adherence and adverse effects throughout follow-up

Twenty-four women reported missing pills during at least one interval of follow-up: 20 missed only 1–2 active pills and four missed 3–5 active pills over at least one interval of follow-up. Reasons for missed pills included forgetting/travelling, losing pills or feeling unwell. Tricycling was practiced by 12 women but not continuously for the full duration of the trial.

Forty women with COCP-exposure answered questions on adverse effects (Supplementary Table 1). Commonly reported effects included breast tenderness (n = 15), irregular bleeding (n = 15) and nausea (n = 8). Nine reported headaches and seven a worsening of acne. “Other” effects were related to mental health (mood change, depression, anxiety and/or sadness).

Women reporting adverse effects of COCP-exposure were contacted to confirm willingness to continue. Five in each group switched arms: 4/5 COCP-users discontinued due to adverse effects (weight gain, mood change, bleeding); five controls requested to commence the COCP, three experienced an unplanned pregnancy but wanted to continue participation. One additional participant received a progesterone-implant during follow-up but remained in the control-arm.

Ninety-two participants submitted baseline requirements, 66 (72%, 95%CI: 61,81) completed the RCT to primary/Amsel-outcome [recurrent BV (n = 30), no recurrence (n = 36)]. Twenty-six women did not return for clinical assessment (attrition rate = 9/100PY, 95%CI: 6,13/100PY), of which 16 returned ≥1 home-pack. Reasons for non-attendance included travel (n = 8), withdrawal due to COCP-related adverse effect (n = 2), or attended General Practice for assessment instead (n = 2). Fourteen did not provide reasons for non-attendance. Eighty-two of 92 women (89%, 95%CI: 81,95) completed the RCT to Secondary/Nugent-outcome, i.e. returned ≥1 pack for microbiological assessment of (1) recurrent-BV (n = 39) or (2) no recurrence (n = 43) by six-months.

### Primary/Amsel-outcome

The primary outcome was assessed by mITT analysis, with BV-recurrence defined as ≥3 Amsel criteria and NS = 4–10 by six-months (Table 3). The overall recurrence rate was 12/100PY (95%CI: 9,18/100PY), with recurrence rates of 14/100PY (95%CI: 9,21/100PY) in the control-arm and 10/100PY (95%CI: 6,19/100PY) in the COCP-arm (p = 0.471, adjusted for lifetime male partners p = 0.469; Supplementary Fig. 1). BV-recurrence rates at Amsel-outcome were similar by Per protocol analysis (PPA), with no difference in rates between arms (p = 0.307, adjusted p = 0.300).

**Table 3:** Recurrence rates analysed as modified-intention-to-treat, per protocol, or by COCP-exposure.

	Modified ITT analysis			
	Primary/Amsel-outcome^a^		Secondary/Nugent-outcome^b^	
	Number, PY	number of cases, rate per 100 PY [95% CI]	Number, PY	number of cases, rate per 100 PY [95% CI]
Total	66, 243	30, 12 [9,18]	82, 278	39, 14 [10,19]
*Control*	40, 146	20, 14 [9,21]	45, 156	23, 15 [10,22]
*COCP*	26, 96	10, 10 [6,19]	37, 123	16, 13 [8,21]
P value^c^		0.471		0.795
P value^d^		0.469		0.803
	**Per protocol analysis**			
	**Primary/Amsel-outcome** ^**e**^		**Secondary/Nugent-outcome** ^**e**^	
	**Number, PY**	**number of cases, rate per 100 PY [95% CI]**	**Number, PY**	**number of cases, rate per 100 PY [95% CI]**
Total	65, 218	27, 14 [9,23]	81, 254	38, 15 [11, 21]
*Control*	40, 132	19, 14 [9,23]	45, 142	23, 16 [11,24]
*COCP*	25, 86	8, 9 [5,19]	36, 112	15, 13 [8,22]
P value^c^		0.307		0.651
P value^d^		0.300		0.608
	**Analysed by COCP-exposure**			
	**BV or no BV by Amsel method** ^**f**^		**BV or no BV by Nugent method** ^**f**^	
	**PY of follow-up** ^**g**^	**number of cases, rate per 100 PY [95% CI]**	**PY of follow-up** ^**g**^	**number of cases, rate per 100 PY [95% CI]**
Total	249	30, 12 [8,17]	278	39, 14 [10,19]
*off COCP*	145	21, 14 [9,22]	158	25, 16 [11,23]
*on COCP*	104	9, 9 [5, 17]	121	14, 12 [7,20]
P value^c^		0.157		0.385
P value^d^		0.149		0.377

PY, perons-years of follow-up; CI, confidence interval.

^a^Modified Intention-to-treat (ITT): completed to primary/Amsel-outcome (BV-recurrence or no recurrence within six-months, measured using Amsel method, censored at LTFU).

^b^Modified Intention-to-treat (ITT): completed to secondary/Nugent-outcome (BV-recurrence or no recurrence within six-months, measured using Nugent method, censored at LTFU).

^c^P value for differences in cumulative BV-recurrence between treatment-arms or COCP-exposure, assessed by Cox regression.

^d^P value for differences in cumulative BV-recurrence between treatment-arms or COCP-exposure, assessed by Cox regression and adjusted for number of lifetime male sexual partners (<15 or ≥15), which was discrepant between randomisation groups at baseline.

^e^Per protocol analysis: provided at least one interval of follow-up for Amsel-outcome or Nugent-outcome, analysis censored at change of treatment-arm (cross-over) or LTFU.

^f^By combined oral contraceptive pill (COCP)-exposure = analysed according to whether “on” or “off” the COCP for any given interval, provided at least one interval of follow-up for Amsel-outcome or Nugent-outcome.

^g^Number of intervals (person-years) participants were exposed to the pill *vs* not exposed. Women who changed arms contributed PY of follow-up to both groups.

### Secondary/Nugent-outcome

The secondary outcome was assessed by mITT analysis with BV endpoint defined as NS = 7–10 or NS < 7 by six-months (Table 3). The overall recurrence rate was 14/100PY (95%CI: 10,19/100PY) with recurrence rates of 15/100PY (95%CI: 10,22/100PY) in controls and 13/100PY (95%CI: 8,21/100PY) in the COCP-arm (p = 0.795). Rates were similar by PPA (p = 0.651) and after adjustment.

### Rate of BV-recurrence by actual pill exposure

We also analysed BV-recurrence according to pill-exposure rather than randomisation (Table 3). By Amsel-outcome, women with COCP-exposure during any given interval had a BV-recurrence rate of 9/100PY (95%CI: 5,17/100PY), which was not significantly lower than in women with no COCP-exposure (14/100PY; 95%CI: 14,22/100PY, p = 0.157, adjusted p = 0.149, Supplementary Fig. 1). Similarly, by Nugent-outcome, COCP-exposure did not result in lower recurrence rates (p = 0.385, adjusted p = 0.377).

We were concerned that randomisation might influence condom-use despite equal counselling. Using logistic regression, condom-use by randomisation-arm was examined, clustering observations by participant. There was no difference in consistent condom-use between arms (Amsel-outcome: Odds ratio[OR] = 1.23, 95%CI: 0.61,2.50, Nugent-outcome: OR = 1.27, 95%CI: 0.67,2.40).

### Characteristics associated with BV-recurrence

The influence of specific characteristics on BV-recurrence in all women at Amsel/Nugent-outcomes was determined using Cox regression adjusting for randomisation (Table 4). Women reporting past BV were more likely to experience recurrence (Amsel: HR = 2.79, 95%CI: 1.06,7.36, p = 0.038; Nugent: HR = 2.23, 95%CI: 1.05, 5.02, p = 0.038). During follow-up, sex with an ongoing partner (any gender from pre-antibiotic treatment) conferred a 2.5–3-fold increased risk of recurrence compared to no sex/sex with a new partner only (Amsel: HR = 2.87, 95%CI: 1.36,6.07, p = 0.006; Nugent: HR = 2.54, 95%CI: 1.34,4.83, p = 0.004). Additionally, increased sex frequency with an ongoing partner increased the recurrence risk, with sex >7-times/month resulting in the highest risk *vs* no sex (Amsel:HR = 3.47, 95%CI: 1.56,7.72, p = 0.002; Nugent: HR = 3.22 95%CI: 1.55, 6.69, p = 0.002). COCP-exposure did not significantly reduce recurrence risk assessed as either *current*-exposure (Amsel: HR = 0.52, 95%CI: 0.16, 1.66) or *any* COCP-exposure over follow-up (Amsel: HR = 0.37, 95%CI: 0.11, 1.28). Condom-use was not associated with recurrence (Amsel:HR = 1.14, 95%CI: 0.55, 2.34). There was also no association between country of birth, education level, current douching practices, sex work, penile-anal sex or receptive oral sex and BV recurrence measured at either study outcome.

**Table 4:** Baseline and time-dependent variables associated with BV-recurrence.

Study population characteristics	Primary/Amsel-outcome (N = 66)			Secondary/Nugent-outcome (N = 82)		
	number of cases, rate per 100 PY (95% CI)	Adjusted HR (95% CI)^a^	p value	number of cases, rate per 100 PY (95% CI)	Adjusted HR (95% CI)^a^	p value
Current age [median, range]	30, 12 (8,17)	1.05 (0.96,1.14)	0.312	39, 14 (10,19)	1.05 (0.98,1.13)	0.181
**Treatment-arm**						
Control	20, 14 (9,21)	1		23, 15 (10,22)	1	
COCP	10, 10 (5,18)	0.71 (0.33,1.52)	0.376	16, 13 (8,21)	0.92 (0.48,1.75)	0.795
***Baseline characteristics***						
**Country of birth**						
Australia/New Zealand	11, 10 (5,17)	1		14, 10 (6,17)	1	
Other	19, 14 (9,22)	1.38 (0.65,2.90)	0.401	25, 18 (12,26)	1.61 (0.83,3.10)	0.160
**Past history of BV**						
No	5, 6 (2,13)	1		8, 8 (4,16)	1	
Yes	25, 16 (11,24)	2.79 (1.06,7.36)	**0.038**	31, 18 (13,26)	2.23 (1.05,5.02)	**0.038**
**Lifetime no. male partners**						
<15	13, 12 (7,21)	1		17, 14 (9,23)	1	
≥15	17, 12 (7,19)	0.98 (0.48, 2.03)	0.964	22, 14 (9,21)	0.97 (0.51, 1.85)	0.938
**Adherence to antibiotics**						
100%	24, 12 (8,18)	1		32, 13 (10,19)	1	
<100%	4, 14 (5,36)	1.06 (0.37,3.09)	0.903	6, 22 (10,48)	1.63 (0.68,3.93)	0.277
***Longitudinal behaviours*** ^**b**^						
**Average no. of cigarettes smoked per week**						
None	17, 13 (8,23)	1		24, 16 (11,24)	1	
1–34	4, 7 (3,18)	0.54 (0.18,1.61)	0.267	6, 10 (4,22)	0.64 (0.26,1.58)	0.333
35 or more	9, 17 (9,32)	1.34 (0.59,3.03)	0.488	8, 13 (6,25)	0.90 (0.40,2.03)	0.801
**Menstrual phase** ^**c**^						
Menses/peri menses	15, 13 (8,22)	1		17, 14 (9,23)	1	
Non-menstrual	15, 11 (7,18)	0.84 (0.41, 1.74)	0.640	22, 14 (9,21)	1.07 (0.57,2.03)	0.824
**Current use of combined oral contraceptive pill**						
No	21, 14 (9,22)	1		25, 16 (11,23)	1	
Yes	9, 9 (5,17)	0.52 (0.16, 1.66)	0.268	14, 12 (7,20)	0.64 (0.24,1.73)	0.376
**Any penile-vaginal sex**						
No	10, 10 (5,19)	1		10, 9 (5,17)	1	
Yes	20, 13 (9,21)	1.24 (0.58,2.67)	0.575	29, 17 (12,24)	1.81 (0.87,3.73)	0.110
**Condom-use for any penile-vaginal sex**						
Always/not practiced	16, 11 (7,18)	1		21, 13 (9,21)	1	
Not always	14, 13 (8,22)	1.14 (0.55,2.34)	0.722	18, 15 (9,24)	1.14 (0.61,2.15)	0.683
**Any new sexual partner**						
None	21, 12 (8,19)	1		25, 14 (9,21)	1	
One or more	9, 11 (6,21)	0.86 (0.39,1.89)	0.713	14, 14 (8,24)	0.90 (0.46,1.78)	0.771
**Penile-vaginal sex with ongoing RSP** ^**d**^						
No	14, 9 (5,14)	1		18, 10 (6,16)	1	
Yes	13, 19 (11,34)	2.45 (1.13,5.30)	**0.024**	19, 23 (15,36)	2.32 (1.21,4.46)	**0.012**
**Sex with ongoing RSP** ^**e**^						
No	14, 8 (5,14)	1		18, 10 (6,15)	1	
Yes	16, 21 (13,34)	2.87 (1.36,6.07)	**0.006**	21, 24 (15,36)	2.54 (1.34,4.83)	**0.004**
**Frequency of penile-vaginal sex with ongoing RSP** ^**f**^						
No penile-vaginal sex	17, 9 (6,15)	1		21, 11 (7,16)	1	
1–7 times per mo	2, 8 (2,32)	0.91 (0.21,3.98)	0.899	4, 13 (5,34)	1.13 (0.38,3.40)	0.827
>7 times per mo	11, 27 (15,48)	3.47 (1.56,7.72)	**0.002**	14, 29 (17,50)	3.22 (1.55,6.69)	**0.002**

Bolded text indicates significant associations at the level p < 0.05.

BV, bacterial vaginosis; CI, confidence interval; HR, hazard ratio; PY, person-years; COCP, combined oral contraceptive pill; MSP, male sexual partner; RSP, regular sexual partner; SP, sexual partner.

^a^All analyses adjusted for randomisation-arm.

^b^Each variable is comprised of behaviours reported longitudinally by participants at each study interval.

^c^Menstrual phase was defined as either (1) menses/peri menstrual: women who were either currently menstruating or were predicted to start menstruating within the next 7 days or (2) non-menstrual: women who were more than 7 days since starting their last menses or who indicated they were currently skipping the sugar pills/menses while using COCP.

^d^Penile-vaginal sex with an ongoing RSP defined as post-treatment sex with the same pre-treatment male RSP.

^e^Sex with an ongoing RSP defined as post-treatment sex with the same pre-treatment RSP, with sex with female RSP defined as having received oral sex and sex with male RSP defined as penile-vaginal sex. For the Amsel-outcome, 28 women (42%) had at least one interval of sex with an ongoing RSP and 32 (48%) at least one interval of sex with a partner that was not the same as pre-treatment. There were nine women who reported sex with a new SP in addition to an ongoing RSP in the same interval and these were counted as sex with an ongoing RSP.

^f^Frequency of sex was cut at the median. NB. Up to 5% of participants may have missing data for some variables; proportions are calculated using available data. There were no significant associations between education level, current sex work, penile-anal sex or receptive oral sex and BV recurrence.

### Multivariate analyses

Variables significantly associated with recurrent BV were included in two multivariate models (Table 5). The first adjusted for treatment-arm and the second for pill-exposure, regardless of randomisation. As condom-use has been shown to have a protective effect against BV^14^ and there were more intervals of inconsistent condom use reported by women with an ongoing RSP compared to women with a new partner (54% *vs* 45%), we included condom-use in both models.

**Table 5:** Longitudinal variables associated with BV-recurrence.

Characteristic (N = 66 Amsels, 82 Nugent)	MODEL 1^a^				MODEL 2^b^			
	Primary/Amsel-outcome		Secondary/Nugent-outcome		Primary/Amsel-outcome		Secondary/Nugent-outcome	
	Adjusted HR (95% CI)	P Value	Adjusted HR (95% CI)	P Value	Adjusted HR (95% CI)	P Value	Adjusted HR (95% CI)	P Value
**Treatment-arm**								
Control	1		1					
COCP	0.63 (0.29, 1.38)	0.252	0.78 (0.40, 1.51)	0.457				
**Past history of BV**								
No	1		1		1		1	
Yes	3.03 (1.14, 8.06)	**0.027**	2.78 (1.22, 6.33)	**0.015**	2.85 (1.34, 6.28)	**0.037**	2.67 (1.18, 6.06)	**0.019**
**Current use of COCP** ^**b**^								
No					1		1	
Yes					0.61 (0.27, 1.36)	0.228	0.78 (0.40, 1.53)	0.477
**Sex with an ongoing RSP** ^**c**^								
No	1		1		1		1	
Yes	3.13 (1.41, 6.94)	**0.005**	2.97 (1.49, 5.93)	**0.002**	2.90 (1.34, 6.28)	**0.007**	2.83 (1.44, 5.54)	**0.002**
**Condom-use for any penile-vaginal sex**								
Always/not practiced	1		1		1		1	
Not Always	0.64 (0.29, 1.40)	0.267	0.66 (0.33, 1.32)	0.236	0.71 (0.33, 1.54)	0.385	0.69 (0.34, 1.38)	0.295

Bolded text indicates significant associations at the level p < 0.05.

BV, bacterial vaginosis; CI, confidence interval; HR, hazard ratio; COCP, combined oral contraceptive pill; RSP, regular sexual partner.

^a^Model 1 adjusted for characteristics associated with BV-recurrence by univariate analysis by treatment-randomisation and condom-use.

^b^Model 2 adjusted for characteristics associated with BV-recurrence by univariate analysis by COCP-exposure and condom-use.

^c^Sex with an ongoing RSP defined as post-treatment sex with the same pre-treatment RSP. Sex with a female RSP is defined as having received oral sex and sex with male RSP is defined as penile-vaginal sex.

NB: Sex with an ongoing partner was correlated with sex frequency, so the former was included as it was most strongly associated with recurrence.

Neither treatment-randomisation nor actual COCP-exposure was associated with recurrence in either model. After adjusting for randomisation, women reporting an ongoing partner had a 3-fold increased risk of recurrence by Amsel/Nugent methods compared to women reporting no sex or a new partner (Amsel:adjusted[A]HR = 3.13, 95%CI: 1.41, 6.94, p = 0.005; Nugent: AHR = 2.97, 95%CI: 1.49, 5.83, p = 0.002), and BV-history conferred a 2.5–3-fold increased risk of BV-recurrence (Amsel: AHR = 3.03, 95%CI: 1.14, 8.06, p = 0.027; Nugent: AHR = 2.78, 95%CI: 1.22, 6.33, p = 0.015). The second model, adjusting for COCP-exposure (regardless of randomisation), replicated these findings; an ongoing sexual partner conferred a 3-fold increased risk of recurrence compared to no sex/new sexual partner (Amsel:AHR = 2.90, 95%CI: 1.34,6.28, p = 0.007) and past BV resulted a 2.5–3-fold increased risk of recurrence (Amsel:AHR = 2.85, 95%CI: 1.14,8.06, p = 0.037). There was no significant association between condom-use and BV-recurrence by either outcome or in either model.

### Predictors of attrition

Attrition was associated with randomisation, with randomisation to COCP-arm associated with a 3-fold increased risk of loss/withdrawal before Amsel-outcome after adjustment (AHR = 3.73, 95%CI: 1.49, 9.39, p = 0.005, Supplementary Table 2). Importantly however, COCP-exposure itself, regardless of randomisation, was not associated with attrition in adjusted analyses (AHR = 1.31; 95%CI: 0.73, 3.47). No other behaviours influenced attrition including partner-type.

## Discussion

In this open-label pilot RCT of the combined oral contraceptive pill in women treated for BV, COCP-exposure did not significantly reduce BV-recurrence measured by either Amsel or Nugent methods. While this finding was likely influenced by the limited sample size and uneven attrition, particularly affecting the COCP-arm, it may also indicate lack of an effect. Interestingly, the effect sizes observed for COCP-exposure on BV-recurrence were of similar magnitude to that reported by meta-analysis (pES = 0.69, 95%CI: 0.59,0.91^13^), suggesting that COCP-exposure may promote a favourable vaginal microbiota following antibiotics. However, multivariate analyses of all participants showed re-exposure to an ongoing regular sexual partner (suggestive of reinfection) conferred a 3-fold increased risk of BV-recurrence and that women with a BV-history (suggestive of persistence) had a 2.5–3-fold increased risk of recurrence. These data indicate that reinfection from an untreated partner and persistence/re-emergence of BV-associated bacteria following treatment are both playing an important role in the pathogenesis of recurrent BV and may have obscured any beneficial effect of hormonal contraceptives on the vaginal microbiota. Our findings provide compelling evidence for the need to evaluate partner treatment and strategies to eradicate BV-associated bacteria including biofilm disruption as adjunctive therapies.

We undertook this pilot to determine whether hormonal contraception following antibiotic therapy reduced BV-recurrence compared to antibiotic therapy alone and to assess the feasibility of the intervention. Women using hormonal contraception have a reduction in BV in a number of observational studies^13,15^, and a recent NuvaRing® trial in 120 women showed improved mean Nugent scores over 3-months^16^. A favourable effect of combined oestrogen-progesterone contraception on the vaginal microbiota may be explained by several mechanisms. Oestrogen increases epithelial glycogen, which is metabolised to lactic acid, with antimicrobial activity against BV-associated bacteria^4,5,17–19^. Immune mechanisms may be regulated by sex hormones directly^20^ or through lactic acid^21,22^. Additionally, pill-exposure reduces menstrual loss and therefore less heme is present in the genital tract, which some BV-associated bacteria require for growth^23,24^. While our trial did not demonstrate a significant effect, it showed a similar > 30% reduced BV-recurrence risk in COCP-users (AHR = 0.63) as reported by meta-analysis (pES = 0.69)^13^. Our findings may be explained by low acceptability and poor retention, particularly in the COCP-arm, or the lack of a biological effect. As consistent condom-use is associated with reduced BV by meta-analysis, it is also possible that the non-significant increase in condom-use in controls may have obscured any beneficial COCP-related effect. Together our data suggest that the benefits of using randomisation as the gold-standard to reduce confounding may be outweighed by the limitations of this design, as higher attrition in women randomised to the COCP impacted on outcomes. Attrition was not associated with COCP-use if women who switched arms were analysed according to actual exposure. We cannot exclude a biologically favourable effect of COCP-exposure on the vaginal microbiota, but the effect is probably modest, and importantly may not be enough to override persistence of BV-associated bacteria or reinfection from partners.

The secondary analyses suggest there are two likely mechanisms involved in post-treatment recurrence: i) BV clearance followed by reinfection from a sexual partner and ii) persistence with re-emergence, perhaps due to specific host factors. Women who have sex with an ongoing RSP post-antibiotics consistently have a 2–3-fold increased risk of BV-recurrence^10,25^, illustrating the robustness of this association. Microbiological data from female and male sexual partners of women with BV demonstrate the effect of a regular partner on the vaginal microbiota. Women with a BV-infected female partner have concordant Nugent-scores^26^ and male partners of women with BV carry BV-associated bacteria^27,28^ on their penile-skin and within their distal urethra. Unfortunately, challenges with recruiting couples, attrition, and limited evidence for the optimal male treatment^29,30^ all pose challenges for researchers undertaking concurrent-partner treatment trials. Clinical trials of male partner treatment are underway in the US (NCT02209519) and Australia (ACTRN12619000196145).

There was a 2–3-fold increased risk of recurrence in women reporting a BV-history, which has been observed previously^10^. Past BV could be associated with BV recurrence for a number of reasons including persistence or re-emergence of BV-associated bacteria and/or biofilm, a host immune-mediated pathway^31^, or as a result of sexual behaviour, i.e. ongoing exposure to the same partner or inconsistent condom use with partners, as mentioned above. Several lines of evidence suggest persistence of BV-associated bacteria and/or biofilm could be occurring post-antibiotic treatment. This may be due to an inability of antibiotics to penetrate the biofilm matrix to target pathogens, antimicrobial resistance^32,33^, ineffective treatment of BV-associated bacteria or high organism loads^34,35^. BV-associated biofilms re-emerge after antibiotics^36,37^, cohesive *Gardnerella vaginalis*, which may be integral to the pathogenesis of BV^38^, is not as susceptible as planktonic *G.vaginalis* to lactic acid^39^, and RNA-sequencing shows gene-regulated processes in *G.vaginalis* biofilm favour bacterial growth^40^.

This pilot trial was challenging to undertake with attrition related to randomisation. Concern about COCP-related adverse effects may have influenced equipoise regarding the COCP prior to recruitment and contributed to attrition from the COCP-arm. It is important to note that attrition was not related to actual COCP-exposure, demonstrating that women want to be able to select their preferred method of contraception and that the COCP is acceptable to women who chose it. Although equal numbers of women switched arms from both randomisation-groups, the three control-arm pregnancies also demonstrate the ethical challenges regarding randomisation to a group that does not contain a hormonal or highly effective method of contraception. Condom-use and emergency contraception were strongly promoted to all participants, as missing pills can also lead to unplanned pregnancies. Although COCP-adherence was self-reported monthly, biomarkers were not collected, so poor adherence may have also undermined findings. Additionally, the trial length (≤six-months) may have been too short to observe a significant benefit on recurrence rates as observational data generally reflects longer periods of COCP-use. Despite the shorter trial length, almost 1/3 did not attend for clinical assessment, which contributed to decreased power in the primary/Amsel-outcome analysis. We used both Amsel and Nugent criteria to comprehensively assess BV and although non-attendance affected the Amsel-outcome, the results between outcomes were consistent. Clearly, approaches to improve retention should be considered. Finally, single-site recruitment may limit generalisability.

## Conclusion

While this RCT of COCP-exposure did not reduce BV-recurrence, the effect size observed for the COCP was in the order of that reported in observational studies, despite limitations with sample size and disproportional attrition. The most striking finding however was that both BV-history and ongoing exposure to an untreated sex partner were associated with post-treatment recurrence. The robustness of these associations illustrates that reinfection from a sexual partner and persistence of BV-associated bacteria are important drivers of post-treatment recurrence, and it is possible that these factors may have overwhelmed any potential benefit from the COCP. This data supports epidemiological evidence that BV-associated bacteria are sexually transmitted. However, data from well-powered RCTs of partner treatment and further evidence of the mechanisms involved is required. Our inability to make any significant inroads into improving BV cure is likely due to the complex pathogenesis of BV and has led researchers to discuss combination approaches including antimicrobials with biofilm disruptors and partner treatment. Hormonal contraception, particularly containing oestrogen, may still support a favourable microbiota following effective treatment strategies and our ongoing microbiota analyses will explore this further. Well-powered RCTs may further determine the effect of the COCP on BV recurrence. But as attrition was associated with randomisation and not pill-use itself, larger observational studies carefully controlling for confounding factors are likely to be more acceptable to women and to yield valuable data that assist in determining whether there is merit in this adjunctive strategy.

## Methods

### Trial design

This was an open-label pilot RCT of COCP-exposure following first-line antibiotics to determine the impact on BV-recurrence, called *Strategies to prevent BV* (SToPBV). Melbourne Sexual Health Centre (Victoria, Australia) attendees with vaginal symptoms received a vaginal examination, pH estimation (Spezialindikator strips pH 2–9, Merck & Co, USA), specimen collection for *C. trachomatis, N. gonorrhoeae* TMA (Hologic Pty Ltd, NSW, Australia) and *T.vaginalis* culture (Trichomonas medium). Women were assessed for BV using both the Amsel method^41^, which documents the presence of a vaginal discharge, a positive amine test, vaginal pH > 4.5 and clue cells present on vaginal smear, and the Nugent scoring (NS) method^42^ was applied by experienced microbiologists, which scores the bacteria present on a vaginal smear, with BV = 7–10, intermediate flora = 4–6 and normal flora = 0–3. Women diagnosed with symptomatic BV, defined as ≥3 Amsel criteria with a Nugent score(NS) = 4–10, were prescribed 7-days of oral metronidazole 400 mg bd or, if contraindicated, 7-days of vaginal clindamycin^7,8^ and referred to trial nurses. Women aged 18–45 with symptomatic BV were eligible if they were willing to comply with protocol requirements and ineligible if they were (i) already using a hormonal contraceptive; (ii) assessed as having contraindications to the COCP using WHO criteria (e.g. focal migraine, history of deep venous thrombosis, hypertension)^43^; (iii) pregnant or wishing to conceive within six months; (iv) concurrently diagnosed with pelvic inflammatory disease or HIV; (v) planning substantial travel; or (vi) did not have an Australian Medicare card or did not have reciprocal Medicare rights from an eligible country. Women were then assessed for equipoise (no objection to randomisation to either arm) and read and signed the Participant Information and Consent Form (informed consent) if willing to participate.

Women were randomised 1:1 to intervention-arm (COCP comprising 21 days of 30 mcg ethinyloestradiol/ 150 mcg levonorgestrel and 7-days inactive pill, to commence the day after antibiotics) or control-arm (continue with current non-hormonal practices). The design was open-label as it was considered unethical to provide women with a placebo COCP. Alternative COCPs could be prescribed if clinically indicated or by participant request and any tricycling (skipping placebo pills to skip or shorten their bleeding period in between packs) was captured in questionnaires. A free-call phone number was provided so participants could report adverse effects, withdraw or for any other enquiries. All women received counselling regarding safe-sex practices and condom-use for STI and pregnancy prevention, and advised about indications for emergency contraception. Following enrolment, participants completed a questionnaire capturing demographics and behaviours, and self-collected a high-vaginal swab for future microbial analysis. Throughout follow-up, participants were contacted for clinic appointments or to prompt specimen collection at home. Reminders were sent with non-responders still able to contribute at the following interval. Follow-up involved questionnaires and self-collection of vaginal swabs and smears for NS-assessment at home at day 8 (d8) and months (M) 1, 2, 4, 5, with an additional clinic/Amsel assessment at M3 and 6. Women with a home NS = 7–10 were recalled to determine if they had symptomatic BV (by Amsel method) upon clinical assessment. Antibiotic adherence was recorded in d8 questionnaires and those in the COCP-arm were asked about adherence, missed pills, acceptability and adverse effects monthly. Participants were reimbursed up to AUD$60.

This trial was prospectively registered with the Australian New Zealand Clinical Trials Registry on the 15/10/2013 (ACTRN12613001147774) with CONSORT reporting^44^. Approval was obtained from the Alfred Hospital (404/13) and University of Melbourne (1340852) Ethics committees, and all research was performed in keeping with the National Statement on Ethical Conduct in Human Research, with informed consent obtained from all participants.

### Study outcomes

The primary outcome was clinically defined BV-recurrence (≥3 Amsel criteria and NS = 4–10) or no recurrence within six-months. No participant with BV-recurrence had a NS < 4. Women were censored from follow-up once either outcome was reached or loss-to-follow-up (LTFU).

The secondary outcome was BV recurrence defined as NS = 7–10 or no recurrence within six months. Women were censored from follow-up once secondary endpoint reached or until LTFU.

### Randomisation, sequence generation, allocation concealment and implementation

A researcher with no clinical role (JSH) generated and held the random number sequence using randomisation block design (size 6) to ensure that allocation of women to the two arms occurred at a similar rate. Numbered, sealed envelopes that concealed allocation were opened consecutively by the research nurse to determine randomisation-group.

### Blinding

Treating clinicians and diagnostic laboratory staff reported Amsel criteria and Nugent score prior to enrolment. Throughout follow-up, one experienced BV trial microbiologist (GF) reported the Nugent score from home samples and MSHC microbiologists reporting the Nugent score, amine and Clue cell criteria from clinical samples were all blinded regarding participant group. The participants, research nurse and treating clinicians were all aware of the allocation as blinding and use of a placebo was deemed unethical and unsafe as: i) women must know if they are at risk of pregnancy and be able to take emergency contraception if indicated, ii) COCP-users experience a regulated menstrual cycle and often mild but distinct initial symptoms such as breast tenderness.

### Sample size

The sample size was estimated for a fully-funded trial using previous six-month recurrence rates (pooled-RR = 0.51, 95%CI: 0.36,0.73)^10,45^. A sample of 266 gave 80% power to detect a 40% reduction in BV-recurrence from 40% in the control-arm to 24% in the COCP-arm (2-alpha = 5%). Assuming 15% LTFU, we aimed to recruit 157 women/arm. The pilot RCT commenced with internal funding, however ongoing funding was not secured and the pilot terminated after 95 recruits (without examining data). Rather than perform retrospective power calculations, we were statistically advised to interpret the likely magnitude of treatment differences using 95% confidence intervals (CIs).

### Data analysis

Analyses were performed using STATA v14.2. The primary analysis was a modified intention-to-treat (mITT) analysis with women analysed as randomised who had ≥1 clinical assessment of BV-recurrence (primary/Amsel-outcome). Secondary analyses included (1) mITT analysis of women analysed as randomised who returned ≥1 microbiological specimen for Nugent scoring (secondary/Nugent-outcome); (2) per-protocol analyses (PPA), which followed women until primary/secondary outcome but censored women who “switched” arms at the point of cross-over; and (3) analyses by COCP-exposure (individuals contributed person-time to intervals “off-COCP” and “on-COCP” if they changed arms) until primary/secondary outcome. Cumulative BV-recurrence rates per 100 person-years (PY) and Poisson 95%CIs were determined for the whole study population and separately by randomisation-arm within six-months, with differences between arms or COCP-exposure groups depicted by Kaplan-Meier survival curves and assessed by Cox regression. Analyses were adjusted for any baseline discrepancies (number of lifetime male partners).

Recurrence rates per 100PY were calculated for baseline and longitudinal characteristics, with Poisson 95%CIs for primary/secondary outcomes. Additional Cox regression analyses of all recruited women were performed to calculate Hazard ratios (HRs) for univariate factors associated with BV-recurrence. Multivariate analyses included covariates associated with recurrence by univariate analysis and either randomisation-arm (Model 1) or COCP-exposure (Model 2).

To inform feasibility, the proportion recruited, COCP-adherence and adverse effects were described. The attrition rate per 100PY until primary/Amsel-outcome was calculated for selected variables, with Poisson 95%CIs. Cox regression assessed factors associated with attrition.

## Supplementary information

Supplementary Material

## Data Availability

The datasets analysed during the current study are not publicly available due to the highly sensitive nature of the questions answered by study participants, which provide extensive detail on participants’ sexual behaviours. The data is required to be securely stored in keeping with Alfred Hospital Ethics requirements. Therefore data is only available upon request by contacting the corresponding author on reasonable request.
