# Supplementary material for: Combined oral contraceptive pill-exposure alone does not reduce the risk of bacterial vaginosis recurrence in a pilot randomised controlled trial
Source: Sci Rep. 2019 Mar 5;9:3555. doi: 10.1038/s41598-019-39879-8 (PMC6401172; doi:10.1038/s41598-019-39879-8)
Supplement: Supplementary file 1 — Supplementary Material [file 41598_2019_39879_MOESM1_ESM.pdf]

Combined oral contraceptive pill-exposure alone does not reduce the risk of bacterial vaginosis recurrence in a pilot randomised controlled trial

Lenka A VODSTRCIL, PhD[1-3]*, Ms Erica PLUMMER[1,2], Christopher K FAIRLEY, MBBS PhD[1,2], Gilda TACHEDJIAN, PhD[4-7], Matthew G LAW, PhD[8], Jane S HOCKING, PhD[3], Ms Karen WORTHINGTON[2], Ms Mieken GRANT[2], Nita OKOKO, BDS[3], Catriona S BRADSHAW, MBBS PhD[1-3]

[1]Central Clinical School, Monash University, Melbourne 3004, Australia

[2]Melbourne Sexual Health Centre, Alfred Hospital, Carlton 3053, Australia

[3]Melbourne School of Population and Global Health, University of Melbourne, Parkville 3010, Australia

[4]Burnet Institute, Melbourne 3004, Australia

[5]Department of Microbiology, Monash University, Clayton 3168, Australia

[6]Department of Microbiology and Immunology, University of Melbourne, at the Peter Doherty Institute of Infection and Immunity, Melbourne 3000, Australia

[7]School of Science, College of Science, Engineering and Health, RMIT University, Melbourne 3000, Australia

[8]Kirby Institute, University of New South Wales, Kensington 2052, Australia

Supplementary Table 1 **Reported adverse effects to the combined oral contraceptive pill (COCP)**

	**Women using COCP (N=40)**	**Intervals of COCP-exposure (N=151)**
Experienced side effect	n, %	n, %
Breast tenderness	15, 38	24, 16
Nausea	8, 20	17, 11
Bleeding or spotting not a regular period	15, 38	23, 15
An increase in headaches	9, 23	11, 7
A worsening of acne	7, 18	14, 9
Other[a]	12, 30	19, 13
Mood change, depression, anxiety, sadness	8	6
Menstruation early, extra, none	2	2
gastrointestinal: cramps, diarrhoea	5	3
weight gain/increased appetite/bloating	4	4

[a] Women could contribute more than one “other” response. One description of an “other” effect was missing and one woman reported “grinding teeth but could be due to school stress”

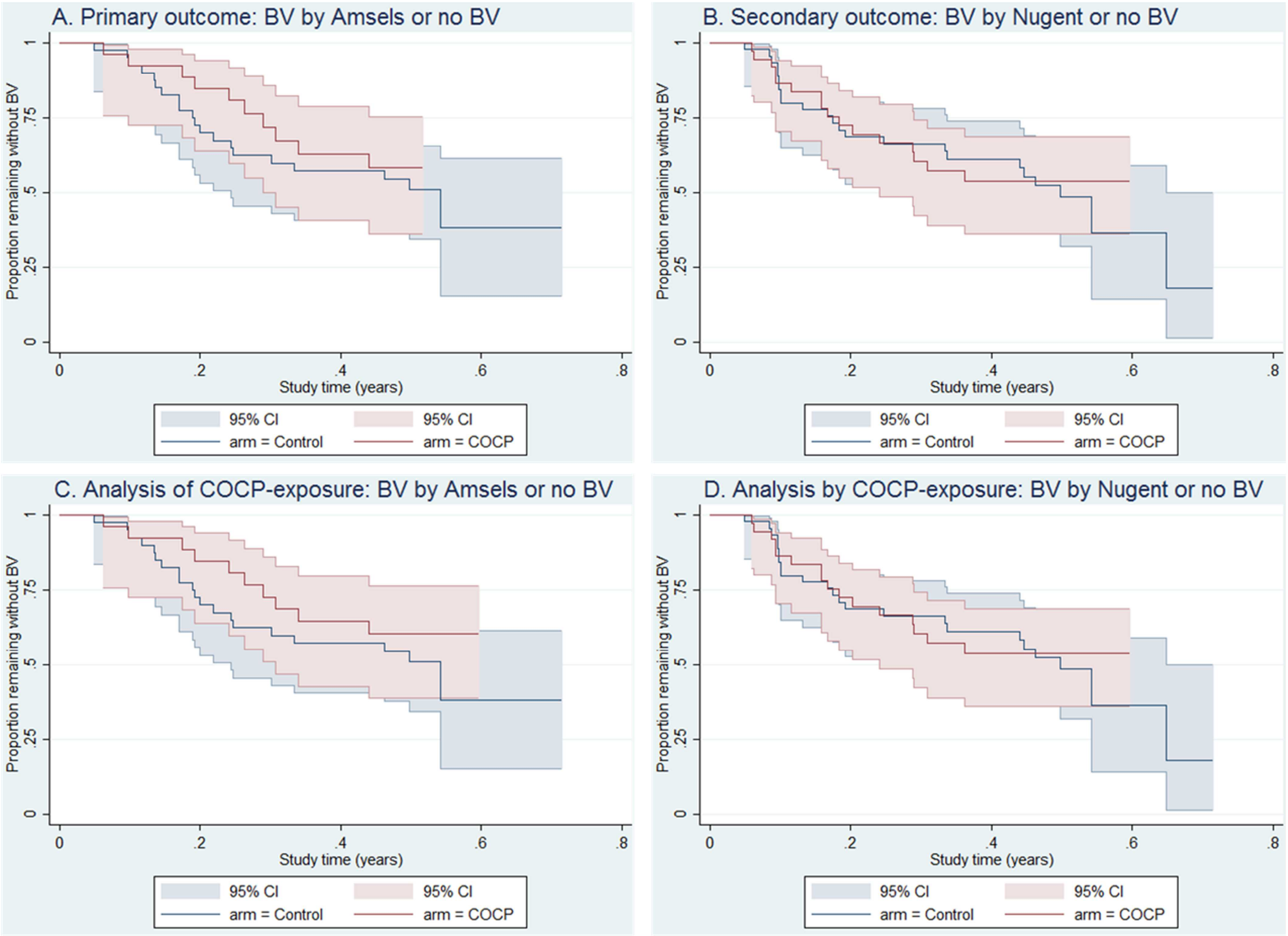

Supplementary Figure 1

Kaplan-Meyer estimates of (A) Time to BV-recurrence or no BV-recurrence as measured at the primary/Amsel-outcome [41], (B) Time to BV-recurrence or no BV-recurrence as measured at the primary/Nugent-outcome [42], (C) Time to BV-recurrence or no BV-recurrence as measured by Amsel method and by COCP-exposure/no exposure, (D) Time to BV-recurrence or no BV-recurrence as measured by Nugent method and by COCP-exposure/no exposure.

Abbreviations: BV, bacterial vaginosis; CI, confidence interval; COCP, combined oral contraceptive pill

Supplementary Table 2 **Characteristics associated with attrition**

				MODEL 1[a]		***MODEL 2***[b]	
Characteristic (N=92)	number LTFU, rate per 100 PY [95% CI]	Unadjusted HR (95% CI)	P Value	Adjusted HR (95% CI)	P Value	Adjusted HR (95% CI)	P Value
Treatment arm							
Control	7, 4 [2, 9]	1		1			
COCP	19, 15 [9, 23]	3.78 (1.51, 9.47)	**0.005**	3.73 (1.49, 9.39)	**0.005**		
Past history of BV							
No	7, 6 [3, 13]	1		1		1	
Yes	19, 10 [7, 16]	1.58 (0.66, 3.78)	0.304	1.63 (0.68, 3.93)	0.277	1.63 (0.68, 3.93)	0.276
Exposure to the COCP[c]							
No	14, 8 [5,14]	1				1	
Yes	12, 9 [5,16]	1.26 (0.57, 2.76)	0.567			1.31 (0.73, 3.47)	0.245
Sex with an ongoing RSP[d]							
No	14, 7 [4, 12]	1		1		1	
Yes	12, 12 [7, 22]	1.57 (0.72, 3.43)	0.255	1.43 (0.65, 3.14)	0.372	1.31 (0.60, 2.89)	0.497

Bolded text indicates significant associations at the level $p < 0.05$.

LTFU, lost-to-follow-up/withdrawn; BV, bacterial vaginosis; CI, confidence interval; HR, hazard ratio; COCP, combined oral contraceptive pill; RSP, regular sexual partner

[a] Model 1 adjusted characteristics associated with BV recurrence by univariate analysis by treatment allocation

[b] Model 2 adjusted characteristics associated with BV recurrence by univariate analysis by COC-exposure

[c] For women LTFU prior to month 1 (M1), variable reflects any COCP-exposure in the last 12 months *vs* no exposure

[d] Sex with an ongoing RSP defined as post-treatment sex with the same pre-treatment partner. Sex with a female RSP is defined as having received oral sex and sex with male RSP is defined as penile-vaginal sex. For women LTFU prior to M1, variable reflects having an RSP at enrolment or not
